# Bile acid-mediated signaling in cholestatic liver diseases

**DOI:** 10.1186/s13578-023-01035-1

**Published:** 2023-04-29

**Authors:** Jing Zeng, Jiangao Fan, Huiping Zhou

**Affiliations:** 1 Department of Microbiology and Immunology, Medical College of Virginia and Richmond VA Medical Center, Central Virginia Veterans Healthcare System, Virginia Commonwealth University, 1220 East Broad Street, MMRB-5044, Richmond, VA 23298-0678 USA; 2 Department of Gastroenterology, Xinhua Hospital, Shanghai Jiao Tong University School of Medicine, Shanghai, 200092 China

**Keywords:** Cholestasis, Bile acids, Bile acid receptors, FXR, TGR5, S1PR2

## Abstract

Chronic cholestatic liver diseases, such as primary biliary cholangitis (PBC) and primary sclerosing cholangitis (PSC), are associated with bile stasis and gradually progress to fibrosis, cirrhosis, and liver failure, which requires liver transplantation. Although ursodeoxycholic acid is effective in slowing the disease progression of PBC, it has limited efficacy in PSC patients. It is challenging to develop effective therapeutic agents due to the limited understanding of disease pathogenesis. During the last decade, numerous studies have demonstrated that disruption of bile acid (BA) metabolism and intrahepatic circulation promotes the progression of cholestatic liver diseases. BAs not only play an essential role in nutrition absorption as detergents but also play an important role in regulating hepatic metabolism and modulating immune responses as key signaling molecules. Several excellent papers have recently reviewed the role of BAs in metabolic liver diseases. This review focuses on BA-mediated signaling in cholestatic liver disease.

## Introduction

Cholestatic liver diseases are characterized by disruption of bile acid (BA) metabolism or bile flow, resulting in the accumulation of BAs in the liver and increased BA concentration in the systemic circulation [1]. Cholestatic liver diseases include primary biliary cholangitis (PBC), primary sclerosing cholangitis (PSC), intrahepatic cholestasis of pregnancy (ICP), progressive familial intrahepatic cholestasis (PFIC) and drug-induced cholestasis [2, 3]. Early clinical manifestations may be asymptomatic, with only elevated levels of alkaline phosphatase (ALP) and gamma-glutamyl transpeptidase (GGT). However, as the disease progresses, symptoms, including pruritus, fatigue, and even hyperbilirubinemia, may occur. Most patients will ultimately need liver transplantation as they develop progressive liver fibrosis, cirrhosis, and liver failure [4–7]. The incidence and prevalence of cholestatic liver diseases have increased globally over the past two decades, and cholestatic liver diseases remain an important public health issue. There is an unmet need to develop effective treatments.

BAs are exclusively synthesized from cholesterol in hepatocytes and stored in the gallbladder as the major components of bile. Maintenance of enterohepatic BA circulation is important not only for nutrient absorption in the intestine but also for hepatic metabolism [1]. BAs can be highly toxic if accumulated in high concentrations in the liver and other tissues due to their amphiphilic structures. The so-called BA pool refers to the total amount of BAs in the enterohepatic circulation, which includes all the BAs in the liver, gallbladder, and intestine. The composition of the BA pool is dynamic and complex [8]. The hydrophobicity of BAs is correlated to their toxicity. BAs are also called steroid acids and act as signaling molecules to regulate metabolic processes by activating nuclear receptors (NRs) and G protein-coupled (GPCRs) [9, 10]. Since the discovery of the first BA-activated NR, the Farnesoid X Receptor (FXR), the physiological and pathological functions of BAs as key signaling molecules have been extensively studied. Identification of BA-activated GPCRs further expanded the BA research field and significantly improved the current understanding by which BAs regulate various physiological and pathological processes. The role of BAs in metabolic diseases has been recently reviewed [10, 11]. Therefore, this review will focus on the current understanding of BAs and BA-mediated signaling pathways in cholestatic liver diseases.

## BA synthesis, metabolism, and circulation

### BA synthesis

**Fig.1:**
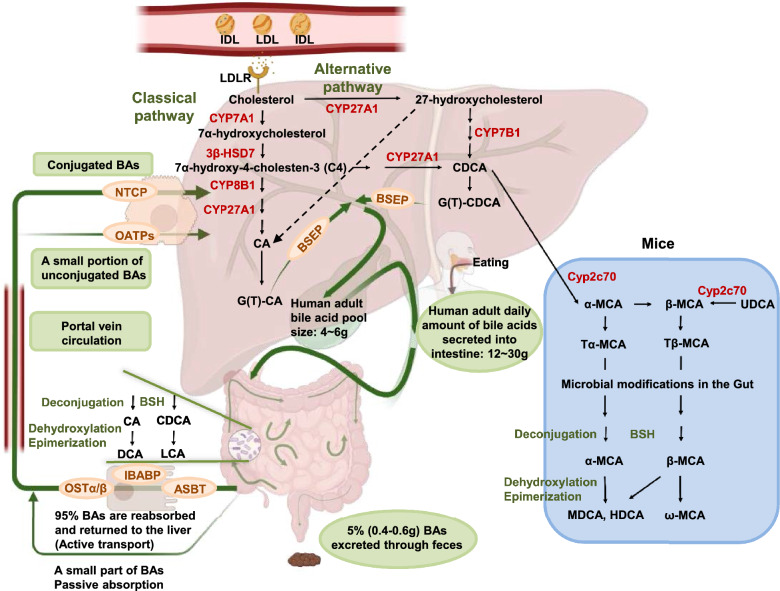
Synthetic pathways of bile acids and enterohepatic bile acid circulation. LDL, low-density lipoprotein; LDLR, low-density lipoprotein receptor; NTCP, Na + -dependent taurocholic acid co-transporting polypeptide; OATP, organic anion-transporting polypeptides; BSEP, bile salt export pump; ASBT, apical sodium-dependent BA transporter; BSH, bile salt hydrolase; IBABP, ileal BA-binding protein; CA, cholic acid; CDCA, chenodeoxycholic acid; OSTα/β, organic solute transporters α and β; MCA, muricholic acid; UDCA, 3α, 7β-dihydroxy5β-cholic acid; MDCA, murine deoxycholic acid; HDCA, hyodeoxycholic acid

BAs are synthesized from cholesterol in hepatocytes, and the liver is the only organ with all the enzymes needed to synthesize BAs exist (Fig. 1). BA synthesis is the main pathway for cholesterol catabolism, with approximately 500 mg of cholesterol converted to BAs per day in adults [12]. Two main pathways have been well characterized in BA synthesis: the classical pathway and the alternative pathway [13]. The classical pathway is also called the "neutral" pathway due to the forming of neutral intermediate metabolites in the process, accounting for the majority (~ 90%) of total BA synthesis. In this pathway, cholesterol is catalyzed first by the rate-limiting enzyme cholesterol 7α-hydroxylase (CYP7A1) to produce 7α-hydroxycholesterol, which is then catalyzed by 3β-hydroxysteroid dehydrogenase 7 (3β-HSD7) in microsomes to generate 7α-hydroxy-4-cholesten-3-one (named C4) [1, 14, 15]. C4 is a common precursor of cholic acid (CA) and chenodeoxycholic acid (CDCA). Therefore, the C4 level reflects the rate of BA synthesis [1, 14]. C4 is catalyzed by sterol 12α-hydroxylase (CYP8B1) and sterol 27-hydroxylase (CYP27A1) to form CA and CDCA. The alternative pathway accounts for only a small part of total BA synthesis in human hepatocytes. It is also called the “acidic” pathway because of the formation of acidic intermediate metabolites during the process. This pathway is initiated by CYP27A1, a mitochondrial enzyme distributed in various tissues and macrophages [16, 17]. Cholesterol is catalyzed by CYP27A1 to generate 27-hydroxycholesterol, which is then converted to 3β-hydroxy-5-cholestenoic acid, and 7-hydroxylation is then performed by oxysterol 7α-hydroxylase (CYP7B1) [1]. This pathway is thought to form CDCA primarily. The BA pool composition of rodents differs from that of humans [18] (Fig. 1). In mouse liver, most CDCA is converted to α-muricholic acid (α-MCA) by cytochrome P450 family 2 subfamily c polypeptide 70 (Cyp2c70). Then the 7α-OH in α-MCA is epimerized to the 7β-OH gene to form β-MCA [13, 19]. MCAs are the major BAs synthesized in mouse liver. The human ortholog cytochrome P450 family 2 subfamily C member 9 (CYP2C9) cannot perform this function, which makes mouse bile more hydrophilic than human bile [20]. In both mice and humans, the 7α-OH in CDCA can be isomerized to 7β-OH to form 3α, 7β-dihydroxy5β-cholic acid (UDCA) [1, 13]. In some pathological conditions, such as cholestatic liver diseases, the classical pathway is inhibited and the alternative pathway is activated as the main pathway for BA synthesis [1]. Mutations in the CYP7A1 gene in adult males cause only mild hypercholesterolemia and early-onset gallstone disease, suggesting that when the classical pathway initiated by CYP7A1 is defective, the alternative BA synthesis pathway is activated to produce BAs [21].

### Enterohepatic BA circulation

Intrahepatic BA circulation is an important physiological process. Upon the formation of primary BAs (CA and CDCA), they undergo detoxification through conjugation with either glycine or taurine [22]. Most primary BAs are conjugated to glycine in humans and taurine in mice. The conjugated BAs cannot penetrate the cell membrane, so an active transport system, ATP-binding cassette (ABC) transporter [mainly bile salt export pump (BSEP)] is needed to mediate the secretion of BAs into the canaliculi, which are small channels between adjacent hepatocytes that ultimately lead to the bile ducts [23]. In certain situations, such as cholestatic liver diseases, the ability of the liver to detoxify BAs may become overwhelmed, leading to a buildup of toxic BAs in the liver and bile ducts. In such cases, some BAs can be reabsorbed by the apical sodium-dependent BA transporter (ASBT), discharged into the periductal capillary plexus via organic solute transporters α and β (OSTα/β) and multidrug resistance-associated protein3 (MRP3), and returned to the hepatocyte, a process known as cholehepatic shunting [24, 25]. This can reduce the overall amount of toxic BAs in the bile ducts and alleviate their harmful effects on the liver. Additionally, cholehepatic shunting can maintain bile flow and enhance bicarbonate-rich choleresis. Previous studies on the function of cholehepatic shunting suggest that stimulate this process may effectively eliminate toxic BAs from the liver and reduce the cholestaic liver injury[26–28]. The three major hepatic lipids (BAs, phosphatidylcholine, and free cholesterol) form mixed micelles and are stored in the gallbladder. Eating stimulates the contraction of the gallbladder to empty its contents to the junction with the duodenum. A small portion of BAs can be absorbed in the duodenum through passive absorption, and about 95% are actively taken up in the ileum via the ASBT at the tip of the brush border of the small intestine and then enter the small intestinal epithelial cells [11, 29]. After binding to ileal BA-binding protein (IBABP), BAs are transported through enterocytes to the basolateral membrane and secreted into the portal vein by OSTα/β [13, 30]. The conjugated BAs in the portal circulation and the systemic circulation are then reabsorbed by hepatocytes via the Na + -dependent taurocholic acid co-transporting polypeptide (NTCP) and secreted into tubules together with newly synthesized BAs through BSEP. A small proportion of unconjugated BAs is reabsorbed by hepatocytes in a Na + -independent manner by organic anion-transporting polypeptides (OATP), including OATP1B1 and OATP1B3.

### Biotransformation of BAs

The gut microbiota consists of a variety of microorganisms. These microbes play key roles in maintaining gut barrier function, regulating metabolic processes, and immune responses [31]. A major function of the gut microbiota is the biotransformation of BAs (Fig. 1). The chemical diversity of BA metabolites is regulated by the deconjugation, dehydrogenation, dehydroxylation, and epimerization of primary BAs in the distal small intestine and colon [32]. Conjugated BAs can activate pancreatic lipase, which in turn releases fatty acid monoglyceride and free fatty acids from triglyceride. The formation of mixed micelles containing fatty acid monoglyceride, fatty acids, cholesterol, and fat-soluble vitamins (A, D, E and K) facilitates their absorption in the small intestine [33]. A few hundred milligrams of BAs escape the ileal absorption and enter the colon, where they are biotransformed by gut bacteria and converted into secondary BAs. More than 50 secondary BAs have been found in human fecal samples [34]. The initial step in the formation of secondary BAs is deconjugation, which is the process of cleaving the C-24N-acylamide of the conjugated BAs and generating unconjugated BAs and glycine or taurine. This step is mediated by bile salt hydrolase (BSH). Functional BSH is present in all major bacteria in the human gut, including gram-negative Bacteroides and gram-positive Lactobacilli and Clostridium [32, 35]. Changes in the gut microbiota also alter BSH expression levels, thereby affecting the composition of the host BA pool [36]. Considering that only conjugated BAs can be efficiently reabsorbed by active transports in the ileum, microbial metabolism can alter intestinal reabsorption of BAs. Therefore, bacterial overgrowth in the small intestine is an important contributor to intestinal BA malabsorption [37]. Unconjugated BAs can pass through the intestinal barrier by passive diffusion or be further modified by the gut microbiome. The primary BAs, CA and CDCA, are oxidized and subsequently 7α-dehydroxylated by specific anaerobic gut bacteria to form secondary BAs, deoxycholic acid (DCA) and lithocholic acid (LCA), respectively [38]. Unlike oxidation and epimerization, only a few anaerobic gut bacteria, about 0.0001% of the gut microbiome belonging to the genus Clostridium, can perform 7α-dehydroxylation [34, 38]. In the human gut, DCA is mainly produced by CA, and LCA and UDCA are produced by CDCA. DCA and a small part of LCA are passively absorbed from the colon into the portal vein. BAs returned from the gut include conjugated BAs as well as unconjugated primary and secondary BAs. In the mice, Tα-MCA and Tβ-MCA are unconjugated by BSH to form α-MCA and β-MCA. α-MCA is further converted to murine deoxycholic acid (MDCA) and hyodeoxycholic acid (HDCA), and β-MCA is converted to ω-MCA. Although MDCA and HDCA can be synthesized from LCA through cytochrome P450 family 3, subfamily a (Cyp3a), the gut bacteria-mediated transformation of α-MCA is the primary source of MDCA and HDCA [39]. And secondary BAs can be converted back to primary BAs by cytochrome P450, family 2, subfamily a, polypeptide 12 (Cyp2a12) in mice [39].

## BAs in cholestatic liver diseases

### Cholangiocyte proliferation

**Fig.2:**
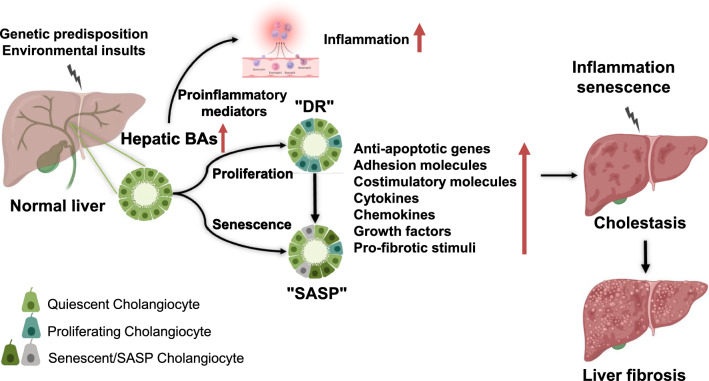
Bile acid-mediated regulation of cholangiocyte proliferation and senescence in the pathogenesis of cholestatic liver diseases. DR, ductular reaction, SASP, senescence-associated secretory phenotype

BA secretion can be impaired in various liver diseases, especially cholestatic liver diseases. Under cholestatic conditions, BAs accumulate in the liver resulting in fewer bile constituents reaching the duodenum. The elevated hepatic BAs will disrupt the tight junctions of biliary epithelial cells (cholangiocytes), leading to bile leakage in the periductal area, which initiates the inflammatory and fibrotic response (Fig. 2). Cholangiocyte proliferation and periportal fibrosis would occur after the accumulation of BAs [40]. It has been reported that TCA could stimulate cholangiocyte proliferation [41].

Cholangiocyte proliferation, also known as the "ductular reaction (DR)," is an adaptive response of cholangiocytes after cholestatic liver injury [42–44]. DR refers to the fact that cholangiocytes become reactive and adopt a neuroendocrine-like phenotype after cholestatic liver injury [45]. This neuroendocrine-like phenotype allows cholangiocytes to secrete in an autocrine and paracrine way in responding to many hormones, neuropeptides, and neurotransmitters [45–47]. Studies have shown that proliferating cholangiocytes express many anti-apoptotic genes, adhesion molecules, costimulatory molecules, cytokines, chemokines, growth factors, and pro-fibrotic stimuli. These factors have both autocrine and paracrine effects on the activation, migration, and proliferation of myofibroblasts [47, 48]. In rodents, DR can be induced by BA feeding and bile duct ligation (BDL) [42] as well as different growth factors and inflammatory cytokines, such as epidermal growth factor (EGF) and vascular endothelial growth factor (VEGF), interleukin-6 (IL-6), IL-1 and tumor necrosis factor α (TNFα) [49, 50]. Early DR may lead to the regression of biliary damage but also can lead to biliary fibrosis if in the presence of persistent inflammation [51, 52]. Ultimately, DR may lead to changes in the cell cycle, senescence, apoptosis, reduction of ducts, mesenchymal infiltration, and sometimes malignant transformation. Therefore, DR is suggested to be the "pacemaker of portal fibrosis" because of the close relationship between cholangiocyte proliferation and fibrosis [48]. Treatments that reduce DR may also reduce the secretion of cytokines, chemokines, and other factors that drive liver fibrosis in cholestatic liver diseases [45]. More research is needed to identify the critical pathways responsible for the DR-associated progression of cholestatic liver diseases.

### Cholangiocyte senescence

The response of cholangiocytes to the injury caused by the elevated levels of BAs is heterogeneous (Fig. 2). Cellular senescence is a pathophysiological state in which proliferating cells enter cell cycle arrest following DNA damage and other stresses [53]. BAs have been identified as potent inducers of cellular senescence [54, 55]. Senescent cholangiocytes exhibit unique phenotypic characteristics, including resistance to apoptosis and a senescence-associated secretory phenotype (SASP) [55, 56]. SASP is a cellular phenotype characterized by increased secretion of proinflammatory cytokines and chemokines, growth factors, metalloproteinases, and extracellular vesicles [57, 58]. SASP has been reported to activate the immune response and recruit immune cells to affected peribiliary areas in PBC [55]. It is worth mentioning that cholangiocyte senescence was first described in the end-stage of PSC patients [59]. To further elucidate the role of cholangiocyte senescence in other stages of PSC, Cazzagon et al*.* recruited 35 PSC patients in a longitudinal study and found that cholangiocyte senescence was present in all stages of PSC. The degree of cholangiocyte senescence is correlated to the histological and clinical severity and disease outcome of PSC [60]. Another study also showed that cholangiocyte senescence directly promoted fibro-inflammatory responses around the bile ducts, which exacerbated the damage and impaired liver regeneration [61]. Cholangiocyte senescence is considered a key pathogenic process in cholestatic disease progression [56, 62, 63]. One potential mechanism is the persistent secretion of fibro-inflammatory mediators through SASP [53]. The work of Barron-Millar et al. highlights the importance of cholangiocyte senescence in the pathogenesis of PBC. It identifies novel prognostic factors that can be used in developing new therapeutic strategies [63]. Recent studies in multidrug-resistance protein 2 knockout (Mdr2^−/−^) mice have shown that a reduction in the number of senescent cholangiocytes represents a potential therapeutic strategy for cholestatic liver injury [64–66].

### Inflammation

It is becoming increasingly clear that BAs represent a major trigger of inflammation in cholestatic liver injury. Allen et al*.* suggested that BAs might induce liver injury by activating an inflammatory response in hepatocytes [67]. Inflammation is a fundamental feature of chronic liver diseases and an important contributing factor to liver fibrosis. Signals from damaged cells, such as ROS, can activate inflammatory cells, including macrophages, lymphocytes, and NK cells et al*.* [68]. These signals from damaged cells and pathogens are called damage-associated molecular patterns (DAMPs) and pathogen-associated molecular patterns (PAMPs), respectively. The core of cholestatic liver diseases is cholangitis, which also suggests direct or indirect damage to cholangiocytes caused by BAs. BAs can stimulate the production of inflammatory mediators, including cytokines, chemokines, and adhesion molecules [67]. Interestingly, cholangiocytes can secrete inflammatory mediators to induce neutrophil activation in response to DAMPs and PAMPs [69–71]. More efforts are needed to understand the complex mechanisms by which inflammation promotes cholestasis liver injury.

### Targeting the BA-mediated signaling pathways as potential therapeutics for cholestatic liver diseases

Since the discovery of the NR, FXR, as a BA receptor in 1999, extensive studies have supported that BAs are essential signaling molecules regulating hepatic metabolism [40, 72–74]. Identification of GPCRs activated by BAs further expanded the field of BA research. BA homeostasis is co-regulated by specific receptors and transporters in the liver and gut [75, 76]. Growing evidence suggests that BA-mediated signaling pathways are involved in cholestatic liver injury, making BA receptors attractive therapeutic targets for cholestatic liver diseases [23, 26, 28].

### Nuclear receptors

NRs are a family of ligand-activated transcription factors that bind to a wide range of natural and synthetic ligands to regulate the development, homeostasis, and metabolism in organisms [77]. BA-activated NRs mainly include FXR, the pregnane X receptor (PXR, also known as NR1I2), the constitutive androstane receptor (CAR, also known as NR1I3), and the vitamin D receptor (VDR) [40, 75].

#### FXR

**Fig.3:**
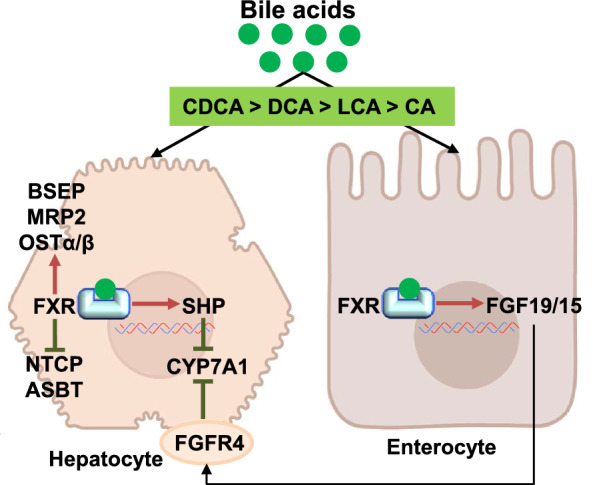
Bile acid-mediated activation of FXR. CDCA, chenodeoxycholic acid; DCA, deoxycholic acid; LCA, lithocholic acid; CA, cholic acid; FXR, Farnesoid X Receptor; SHP, small heterodimer partner; CYP7A1, cholesterol 7α-hydroxylase; FGF, fibroblast growth factor; FGFR4, FGF receptor 4; NTCP, Na + -dependent taurocholic acid co-transporting polypeptide; ASBT, apical sodium-dependent BA transporter; BSEP, bile salt export pump; MRP2, multidrug resistance-associated protein2; OSTα/β, organic solute transporters α and β

FXR, the transcription product of NR1H4, was first discovered by Forman et al*.* in 1995 [78]. It is expressed in the liver, intestine, kidney, adrenal gland, and ovary among which it is highly expressed in the liver and intestine. In the liver, FXR is mainly expressed in cholangiocytes and hepatocytes [13]. In 1999, three groups simultaneously identified BA as the natural ligand for FXR [72–74]. FXR is activated by unconjugated BAs. The potency of BAs in activating FXR varies, with CDCA being the highest, followed by DCA, LCA, and CA [79] (Fig. 3). FXR regulates BA homeostasis in a tissue-specific manner [80]. It should be mentioned here that UDCA, especially glycine-conjugated, does not appear to activate FXR [81], but inhibits FXR [82]. In hepatocytes, FXR activation can induce the expression of the small heterodimer partner (SHP), an atypical member of the NR family that lacks a DNA-binding domain and an inhibitor of CYP7A1 expression, to negatively regulate BA synthesis [83–85]. In the ileum, FXR activation induces expression of the intestinal hormone fibroblast growth factor (FGF) 15/19 (FGF15 in mice and FGF19 in humans), which is secreted as a hormone into the portal circulation. FGF15/19 binds to FGF receptor 4 (FGFR4) on the surface of hepatocytes, inhibiting hepatic CYP7A1 gene transcription through a Jun N-terminal kinase-dependent pathway [12, 86, 87]. Furthermore, FGF15/19 leads to the filling of the gallbladder with bile by regulating the relaxation of the smooth muscle of the gallbladder. FXR activation in the ileum is recognized to play a more important role than the SHP-induced pathway in suppressing hepatic CYP7A1 expression [88]. Activated FXR also prevents BAs accumulation in hepatocytes by inhibiting the uptake by hepatocytes and promoting BAs secretion by directly regulating the expression of human hepatic and intestinal BA transporters, including upregulating BAs efflux transporters BSEP, MRP2, and OSTα/β [89–91], and downregulating the expression of BAs uptake transporters NTCP and ASBT [92]. Overall, FXR can regulate the enterohepatic circulation of BAs and prevent the toxic effects of detergent BAs on hepatocytes and cholangiocytes.

Several published studies have shown that semisynthetic and nonsteroidal agonists of FXR are able to reduce liver inflammation and fibrosis in animal models of cholestasis [93–95]. The synthetic BA derivative obeticholic acid (OCA) is a potent and selective FXR agonist with anti-cholestatic effects [96, 97]. In human clinical studies (Table 1), OCA significantly reduced ALP and GGT, compared with placebo, in PBC patients who had inadequate responses to UDCA [98]. OCA monotherapy significantly improved the long-term clinical outcomes of PBC [99, 100]. In animal studies, OCA increases insulin sensitivity, inhibits gluconeogenesis and adipogenesis, and has anti-inflammatory and anti-fibrotic properties [101, 102]. However, the most common side effect of OCA is a dose-dependent development of itching [98, 99]. In addition to OCA, other FXR agonists are emerging as potential treatments for cholestatic liver diseases (Table 1). Tropifexor (LJN452) improved markers of cholestasis and showed an acceptable safety-tolerability profile, supporting its further clinical development for PBC [103]. Cilofexor (GS-9674) was also well tolerated and attenuated cholestasis in PSC patients in the phase 2 study [104, 105]. Meanwhile, EDP-305, a novel FXR agonist, reduced fibrosis progression in rat BDL model and had also finished a phase 2 clinical trial [106]. As previously mentioned, FXR activation results in the upregulation of FGF15/19 and the downregulation of NTCP and ASBT. Recently, many FGF19 analogs and ASBT inhibitors have been developed. Some of them are currently in various stages of clinical trials for cholestatic liver diseases (Table 2). Aldafermin (NGM282), an FGF19 analog, showed potent suppression of hydrophobic bile acids across metabolic and cholestatic liver diseases in the phase 2 study [107]. On the other hand, Odevixibat (A4250), an ASBT inhibitor, shown to reduce the pruritus and the levels of serum BAs, and was also generally well tolerated in children with PFIC1/2 in a phase 3 study [108]. Linerixibat (GSK2330672), another ASBT inhibitor, demonstrated efficacy in reducing pruritus severity in PBC, but the long-term use of this drug may be limited with the common adverse event of diarrhea, which needs more attention in future studies [109, 110]. Meanwhile, Maralixibat (LUM001) also led to rapid and sustained reductions in serum BA levels, as well as reductions in pruritus in PFIC patients [111]. It was the first agent to show durable and clinically meaningful improvements in cholestasis in children with Alagille Syndrome (ALGS), which might represent a new treatment paradigm. However, it also has gastrointestinal-related side effects [112]. Notably, patients with chronic and advanced cholestasis often are at higher risk of developing hepatocellular carcinoma and cholangiocarcinoma, which may be closely related to the downregulation of hepatic FXR. Increased hepatocellular carcinoma in Fxr^−/−^ mice is associated with elevated serum TCA and activation of c-Myc [113]. Overall, it is important to note that FXR agonists may cause side effects such as diarrhea, abdominal pain, and nausea. Additionally, the long-term safety of FXR agonists remains uncertain. While FXR agonists have shown promise in reducing bile acid accumulation and improving liver function, their efficacy may be limited in advanced stages of cholestatic liver disease. Therefore, further research is necessary to fully evaluate the safety and efficacy of FXR agonists in this patient population.

**Table 1:** The major clinical trials of FXR agonists for cholestatic liver diseases

Drug Name	Indication	Clinical Trials No	Start Year	Status	Sponsor
OCA (obeticholic acid), 6-ECDCA (6-ethyl-chenodeoxycholic acid), or INT-747	PBC	NCT00570765	2008	Phase 2 (Completed)	Intercept Pharmaceuticals
OCA [100]	PBC	NCT01473524	2012	Phase 3 (Completed)	Intercept Pharmaceuticals
OCA	PBC	NCT02308111	2014	Phase 4 [Terminated (Due to the lack of feasibility for this post-marketing study as designed)]	Intercept Pharmaceuticals
OCA [97]	PSC	NCT02177136	2015	Phase 2 (Completed)	Intercept Pharmaceuticals
OCA	Pediatric Subjects With Biliary Atresia	NCT05321524	2015	Phase 2 (Active, not recruiting)	Intercept Pharmaceuticals
OCA	PBC	NCT03633227	2018	Phase 4 (Terminated (Due to Ocaliva (obeticholic acid) US labeling update, the sponsor decided to terminate the study))	Intercept Pharmaceuticals
Tropifexor (LJN452) [103]	PBC	NCT02516605	2015	Phase 2 (Completed)	Novartis Pharmaceuticals
Cilofexor (GS-9674)	PBC	NCT02943447	2016	Phase 2 (Terminated because of the availability of alternate therapies for PBC)	Gilead Sciences
Cilofexor (GS-9674)	PSC	NCT02808312	2016	Phase 1 (Completed)	Gilead Sciences
Cilofexor (GS-9674) [104]	PSC	NCT02943460	2016	Phase 2 (Completed)	Gilead Sciences
Cilofexor (GS-9674)	PSC	NCT03890120	2019	Phase 3 [Terminated (Following recommendation of the external Data Monitoring Committee, after it reviewed the results of a planned interim futility analysis.)] (Updated on January 23, 2023)	Gilead Sciences
EDP-305	PBC	NCT03394924	2017	Phase 2 (Completed)	Enanta Pharmaceuticals, Inc
TQA3526	PBC	NCT04278820	2020	Phase 2 (Unknown)	Chia Tai Tianqing Pharmaceutical Group Co., Ltd
ASC42	PBC	NCT05190523	2022	Phase 2 (Recruiting)	Gannex Pharma Co., Ltd
Linafexor (CS0159)	PSC	NCT05082779	2021	Phase 1 (Completed)	Cascade Pharmaceuticals, Inc
Linafexor (CS0159)	PBC	NCT05624294	2022	Phase 1 (Recruiting)	Cascade Pharmaceuticals, Inc

**Table 2:** The major clinical trials of FGF19 analogs and ASBT Inhibitors for cholestatic liver diseases

Drug Name	Indication	Targets and Mechanism	Clinical Trials No	Start Year	Status	Sponsor
Aldafermin (NGM282)	PBC	FGFR4 (FGF19 analogue)	NCT02026401	2014	Phase 2 (Completed)	NGM Biopharmaceuticals, Inc
Aldafermin (NGM282) [107]	PSC	FGFR4 (FGF19 analogue)	NCT02704364	2016	Phase 2 (Completed)	NGM Biopharmaceuticals, Inc
Odevixibat (A4250)	PBC Pruritus	ASBT (Inhibitor)	NCT02360852	2015	Phase 2 [Terminated ((Expected) side effects)]	Sahlgrenska University Hospital, Sweden
Odevixibat (A4250)	Pediatric Cholestasis	ASBT (Inhibitor)	NCT02630875	2015	Phase 2 (Completed)	Albireo
Odevixibat (A4250) [108]	Children With PFIC1/2	ASBT (Inhibitor)	NCT03566238	2018	Phase 3 (Completed)	Albireo
Linerixibat (GSK2330672) [109]	PBC Pruritus	ASBT (Inhibitor)	NCT01899703	2014	Phase 2a (Completed)	GlaxoSmithKline
Linerixibat (GSK2330672)	PBC Pruritus	ASBT (Inhibitor)	NCT02801981	2016	Phase 1 (Completed)	GlaxoSmithKline
Linerixibat (GSK2330672) [110]	PBC Pruritus	ASBT (Inhibitor)	NCT02966834	2017	Phase 2b (Completed)	GlaxoSmithKline
Linerixibat (GSK2330672)	PBC Pruritus	ASBT (Inhibitor)	NCT04950127	2021	Phase 3 (Recruiting)	GlaxoSmithKline
Maralixibat (LUM001) [111]	PFIC	ASBT (Inhibitor)	NCT02057718	2014	Phase 2 (Completed)	Mirum Pharmaceuticals, Inc
Maralixibat (LUM001)	PSC	ASBT (Inhibitor)	NCT02061540	2014	Phase 2 (Completed)	Mirum Pharmaceuticals, Inc
Maralixibat (LUM001)	PFIC	ASBT (Inhibitor)	NCT03905330	2019	Phase 3 (Completed)	Mirum Pharmaceuticals, Inc
Maralixibat (LUM001) [112]	ALGS	ASBT (Inhibitor)	NCT02160782	2014	Phase 2 (Completed)	Mirum Pharmaceuticals, Inc
Maralixibat (LUM001)	PFIC; ALGS; CLD	ASBT (Inhibitor)	NCT04729751	2021	Phase 2 (Recruiting)	Mirum Pharmaceuticals, Inc
Volixibat	PSC	ASBT (Inhibitor)	NCT04663308	2020	Phase 2 (Recruiting)	Mirum Pharmaceuticals, Inc
Volixibat	ICP	ASBT (Inhibitor)	NCT04718961	2021	Phase 2 (Active, not recruiting)	Mirum Pharmaceuticals, Inc
A3907	PSC	ASBT (Inhibitor)	NCT05642468	2023	Phase 2 (Recruiting)	Albireo
Maralixibat chloride (TAK-625)	PFIC	ASBT (Inhibitor)	NCT05543187	2023	Phase 3 (Recruiting)	Takeda
Maralixibat chloride (TAK-625)	ALGS	ASBT (Inhibitor)	NCT05543174	2023	Phase 3 (Recruiting)	Takeda

#### PXR

Another BA-activated NR, PXR, is highly expressed in the small intestine and hepatocytes [114]. PXR is mainly activated by LCA (both free and conjugated) and DCA. PXR plays an essential role in the degradation and clearance of toxins [115]. PXR signaling is known to regulate the expression of drug-metabolizing enzymes and transporters (DMETs) to facilitate the metabolism, transport, and clearance of xenobiotics [116]. In addition to DMET regulation, PXR is also involved in energy homeostasis, endobiotic metabolism (e.g., BAs, glucose, and lipids), and inflammation regulation [116, 117]. Activated PXR promotes the 6-hydroxylation and increases the water solubility of LCA by inducing the expression of CYP3A [118, 119]. PXR is positively regulated by FXR, and the two receptors act synergistically to ensure BA homeostasis [120]. PXR activation also inhibits hepatic CYP7A1. Recently, Huang et al*.* reported that a lathyrane diterpenoid (5/11/3 ring system), a highly selective agonist of human PXR, exerted its anti-cholestatic effect via activation of the PXR pathway, accelerating the detoxification of toxic BAs and promoting liver regeneration in LCA-induced cholestasis mouse model [121]. While PXR agonists have shown promise in preclinical studies, clinical trials have not yet demonstrated significant efficacy in treating cholestatic liver diseases. Although the discovery of novel PXR agonists holds potential value in the development of anti-cholestasis drugs, further research is necessary to determine their efficacy and long-term safety in clinical settings.

#### VDR

VDR is expressed in both biliary epithelial cells in the liver and the intestine. VDR is nearly ten times more sensitive to LCA than PXR. Activation of VDR protected hepatocytes from cholestatic injury by inhibiting the expression of genes involved in bile acid metabolism and transport [122]. Deletion of VDR promoted cholestatic liver injury by diminishing bile duct integrity in mice [123]. VDR deletion in the intestine can reduce the expression of CYP3A and inhibit the metabolism of LCA [124]. At the same time, VDR deletion in the intestine can indirectly upregulate the expression of BA transporters resulting in promoting enterohepatic circulation and more BAs to the liver, which in turn leads to hepatic cholestasis and liver injury [125]. Previous studies showed that the VDR–YAP axis promotes cholangiocyte proliferation and enhances adaptive bile duct remodeling, alleviating cholestatic liver injury in BDL mice [126]. VDR activation mitigated cholestatic liver injury by reducing autophagy-dependent hepatocyte apoptosis and suppressing the activation of the ROS-dependent ERK/p38MAPK pathway [127]. While modulating VDR activity may be a potential target for treating cholestatic liver diseases, it is important to note that VDR activity can affect calcium metabolism and influence blood calcium levels. This could be particularly concerning in patients with liver diseases. Thus, more research is needed to fully understand the efficacy, safety, and optimal dosing regimens of VDR agonists before they can be considered a viable treatment option.

### G-protein-linked receptors (GPCRs)

The seven transmembrane GPCRs are the most prominent family of membrane proteins and are responsible for most signal transduction from extracellular to intracellular. GPCRs are also the most diverse class of transmembrane proteins, which can sense various environmental stimuli, such as light, lipids, sugars and proteins. Takeda G protein-coupled receptor 5 (TGR5, also known as GPBAR1 or M-BAR), is the first BA-activated GPCR identified in macrophages [76]. During the last decade, several studies also reported that sphingosine-1-phosphate receptor 2 (S1PR2) and the muscarinic receptors were also activated by BAs [128, 129]. BA-mediated activation of GPCRs induces the activation of different downstream signaling pathways based on the coupling of different G proteins in a cell-type-specific manner. GPCRs represent the most important drug targets, and more than 700 FDA-approved drugs target GPCRs [130]. Understanding BA-mediated activation of GPCRs will provide critical information for developing novel therapeutic agents for cholestatic liver disease [131].

#### TGR5

**Fig.4:**
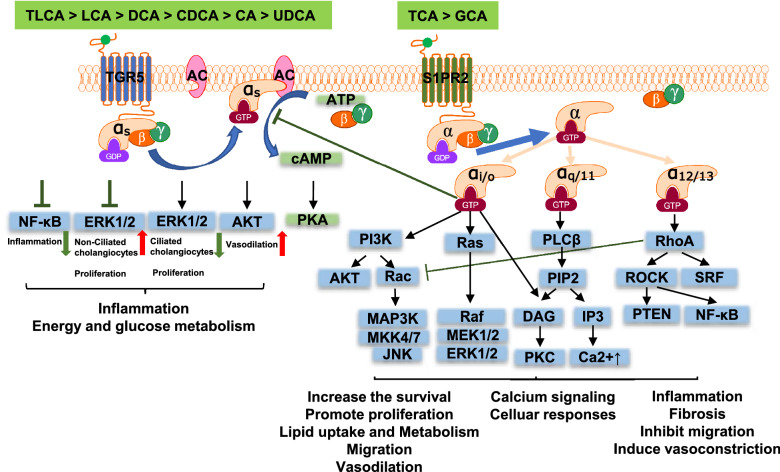
Bile acid-activated GPCRs. TLCA, taurolithocholic acid; LCA, lithocholic acid; DCA, deoxycholic acid; CDCA, chenodeoxycholic acid; CA, cholic acid; UDCA, 3α, 7β-dihydroxy5β-cholic acid; TCA, taurocholic acid; GCA, glycocholic acid; TGR5, Takeda G protein-coupled receptor 5; GDP, guanine dinucleotide phosphate; GTP, guanine trinucleotide phosphate; ATP, adenosine triphosphate; cAMP, cyclic adenosine phosphate; NF-κB, nuclear transcription factor kappa B; ERK, extracellular signal-regulated kinase; PKA, protein kinase A; S1PR2, sphingosine-1-phosphate receptor 2; EGFR, epidermal growth factor receptor

TGR5 was initially identified in macrophages as the first GPCR activated by BAs [76]. It is widely expressed in various tissues, including the intestine, colon, endocrine glands, adipose tissue, muscles, immune organs, gallbladder, kidney, and liver [132–134]. In the liver, TGR5 is highly expressed in non-parenchymal cells, including hepatic sinusoidal endothelial cells [135], activated hepatic stellate cells (HSCs), and intrahepatic [136] and extrahepatic [137] cholangiocytes, Kupffer cells [138], but not expressed in hepatocytes [49]. TGR5 was mainly activated by secondary BAs with the following rank order: TLCA > LCA > DCA > CDCA > CA > UDCA (Fig. 4) [132, 139]. TGR5 also can be activated by steroid hormones. Activation of TGR5 is mainly coupled to Gα_s,_ resulting in the activation of adenylyl cyclase and the elevation of cAMP levels. It has been reported that TGR5 is coupled to Gα_i_ in ciliated H69 cholangiocytes [136]. TGR5 also activates AKT and ERK signaling pathways and regulates glucose and energy metabolism [140]. In addition, TGR5 has been identified as a negative regulator of liver inflammation via inhibiting NF-κB signaling [128, 140–142]. TGR5 activation can induce cholangiocyte regeneration to maintain the integrity of the biliary tree and control the hydrophobicity of BA pools by stimulating bicarbonate secretion [28, 141, 143]. In the BDL and BA-feeding cholestatic mouse models, TGR5^−/−^ mice appeared to develop more severe inflammation and cholestatic liver injury than WT mice. These studies suggest that TGR5 agonists may be beneficial to prevent cholestatic liver injury [144].

Extensive efforts have been put into developing selective and potent TGR5 agonists in the past decade. The 6α-ethyl-23(S)-methylcholic acid (S-EMCA, INT-777) is the best-known semisynthetic TGR5 agonist. However, TGR5 agonists alone did not improve liver fibrosis in Mdr2^−/−^ mice, and the dual TGR5/FXR agonist (INT-767) reduced liver inflammation and fibrosis, possibly by lowering BA synthesis in an FXR-dependent manner [145]. Simultaneous activation of TGR5 and FXR receptors improves prognosis, which may represent a better therapeutic strategy [131]. Considering the broad expression of TGR5, activation of TGR5 in cholangiocytes and macrophages may be beneficial to reduce cholestatic liver injury and inflammation. However, it will cause unwanted effects in other cells and tissues, such as increased gallstone formation by altering gallbladder motility and promoting cholangiocarcinoma cell proliferation [146, 147]. Another side effect of the TGR5 agonist is pruritus. It is necessary to take this into consideration in the future development of therapeutic agents targeting TGR5.

#### S1PR2

S1PR2 was initially identified as a BA-activated GPCR in primary rodent hepatocytes [128]. S1PR2 is one of five S1PRs originally discovered as endothelial differentiation G protein-coupled receptor 5 (EDG5) [41]. S1PR2 is highly expressed in hepatocytes, cholangiocytes, and immune cells in the liver. It is mainly activated by conjugated primary BA, TCA and GCA. Compared to S1P, the ligand affinity of TCA to S1PR2 is 100 times lower. However, TCA-mediated activation of S1PR2 plays an essential role in regulating hepatic lipid and glucose metabolism [33]. S1PR2 can activate various signaling pathways via coupling with different G-proteins [148](Fig. 4). Our previous studies also reported that the upregulation of S1PR2 expression is associated with cholestatic liver fibrosis [41, 149]. TCA-induced activation of AKT and ERK1/2 signaling pathways via S1PR2 promoted cholangiocarcinoma cell proliferation and invasion [150]. Activation of S1PR2 has also been associated with inflammation and mitochondrial dysfunction [151]. A study reported that S1PR2 deficiency inhibits macrophage proinflammatory activities in apoE-deficient mice [152]. However, this paper was retracted due to data manipulation. Therefore, more rigorous studies are needed to understand the role of S1PR2 in modulating inflammatory response in immune cells. The development of more selective and potent antagonists of S1PR2 is critical to test the therapeutic effects for cholestatic liver diseases.

#### Muscarinic receptor 3 (M3)

The muscarinic receptors (M) are composed of five subtypes, M1-M5, with different tissue distributions and overlapping functions by coupling to similar G proteins [153]. M1 and M3 receptors are activated not only by acetylcholine but also by selected BAs. M3 is located at cholangiocyte cell membrane invaginations [154, 155], which is the primary cholangiocyte receptor for different parasympathetic regulation [156]. TLCA has been reported as an antagonist of M3. TLCA inhibits the acetylcholine-induced increase in inositol phosphate formation and activation of mitogen-activated protein kinase (MAPK) [129]. Acetylcholine is rapidly degraded by acetylcholinesterase upon release. Cholinergic stimulation appears to have pro-proliferative, pro-survival effects on biliary growth. BDL mice undergoing vagotomy showed a decreased biliary mass and M3 expression and increased cholangiocyte apoptosis [157]. PBC patients frequently showed autoantibodies directed against M3 [158]. Previous studies also reported that M3 signaling significantly influenced bile formation, M3^−/−^ increased susceptibility to cholestatic injury, and treatment of Mdr2^−/−^ mice with M3 agonist decreased liver injury [159]. Furthermore, human HSCs also express M receptors, and M3 is upregulated in activated HSCs. HSCs secrete and respond to acetylcholine in an autocrine and paracrine manner to increase their expression of proliferative and fibrotic markers [160]. These findings suggested that M3 could play an important role in etiopathogenesis and may represent a promising novel therapeutic target in cholestatic liver diseases.

## Summary and future direction

As important signaling molecules, BAs play critical roles in regulating enterohepatic bile acid homeostasis, hepatic metabolic function, and immune responses under normal physiological conditions. Disruption of BA-mediated signaling pathways has been closely associated with various liver diseases, including cholestatic liver disease. The differential expression of different BA receptors and dynamic changes in BA composition and levels under cholestatic conditions contribute to disease progression. Understanding the role of individual BA receptor-mediated signaling pathways in different types of cells and tissues under physiological and pathological conditions is critical to developing better therapeutics for cholestatic liver diseases. The therapeutic application of the current available agonists and antagonists of BA receptors is limited due to severe side effects and lack of tissue or/cell type specificity. There is an urgent need to develop tissue- or cell-type-selective agonists or antagonists of BA receptors as potential therapeutics for cholestatic liver diseases.

## Data Availability

Not applicable.

## Abbreviations

BA: Bile acid
PBC: Primary biliary cholangitis
PSC: Primary sclerosing cholangitis
ICP: Intrahepatic cholestasis of pregnancy
PFIC: Progressive familial intrahepatic cholestasis
ALP: Alkaline phosphatase
GGT: Gamma-glutamyl transpeptidase
NR: Nuclear receptor
FXR: Farnesoid X Receptor
CYP7A1: Cholesterol 7α-hydroxylase
3β-HSD7: 3β-Hydroxysteroid dehydrogenase 7
CA: Cholic acid
CDCA: Chenodeoxycholic acid
CYP8B1: Sterol 12α-hydroxylase
CYP27A1: Sterol 27-hydroxylase
CYP7B1: Oxysterol 7α-hydroxylase
α-MCA: α-Muricholic acid
CYP2C70: Cytochrome P450 family 2 subfamily c polypeptide 70
CYP2C9: Cytochrome P450 family 2 subfamily C member 9
UDCA: 3α, 7β-Dihydroxy5β-cholic acid or ursodeoxycholic acid
ABC: ATP-binding cassette
BSEP: Bile salt export pump
ASBT: Apical sodium-dependent BA transporter
OSTα/β: Organic solute transporters α and β
MRP3: Multidrug resistance-associated protein3
IBABP: Ileal BA-binding protein
NTCP: Na + -dependent taurocholic acid co-transporting polypeptide
OATP: Organic anion-transporting polypeptides
BSH: Bile salt hydrolase
DCA: Deoxycholic acid
LCA: Lithocholic acid
MDCA: Murine deoxycholic acid
HDCA: Hyodeoxycholic acid
CYP3a: Cytochrome P450 family 3, subfamily a
CYP2a12: Cytochrome P450, family 2, subfamily a, polypeptide 12
DR: Ductular reaction
BDL: Bile duct ligation
EGF: Epidermal growth factor
VEGF: Vascular endothelial growth factor
IL-6: Interleukin-6
TNFα: Tumor necrosis factor α
SASP: Senescence-associated secretory phenotype
Mdr2^-/-^: Multidrug-resistance protein 2 knockout
DAMPs: Dead cell-associated molecular patterns
PAMPs: Pathogen-associated molecular patterns
PXR: Pregnane X receptor
CAR: Constitutive androstane receptor
VDR: Vitamin D receptor
SHP: Small heterodimer partner
FGF: Fibroblast growth factor
FGFR4: FGF receptor 4
OCA: Obeticholic acid
ALGS: Alagille Syndrome
DMETs: Drug-metabolizing enzymes and transporters
UGT1A1: UDP glucuronosyltransferase family 1 member A1
GPCRs: G protein-coupled receptors
TGR5: Takeda G protein-coupled receptor 5
S1PR2: Sphingosine-1-phosphate receptor 2
cAMP: Cyclic adenosine phosphate
PKA: Protein kinase A
HSC: Hepatic stellate cell
EDG5: Endothelial differentiation G protein-coupled receptor 5
M: Muscarinic receptors
MAPK: Mitogen-activated protein kinase
